# A new direction to understand the life cycle of the Japanese pine sawyer considering the selection strategy of instar pathways

**DOI:** 10.1038/s41598-020-73344-1

**Published:** 2020-10-12

**Authors:** Su Bin Kim, Dong-Soon Kim

**Affiliations:** 1 Majors in Plant Resource Sciences and Environment, College of Applied Life Science, SARI, Jeju National University, Jeju, 63243 Republic of Korea; 2 The Research Institute for Subtropical Agriculture and Biotechnology, Jeju National University, Jeju, Republic of Korea

**Keywords:** Ecology, Zoology

## Abstract

The Japanese pine sawyer, *Monochamus alternatus* Hope (Coleoptera: Cerambycidae), transfers the pine wood nematode, *Bursaphelenchus xylophilus* (Steiner and Buhrer) that causes pine wilt disease (PWD), especially in Asian countries. The key for the control of PWD is primarily focused on vector management. Thus, understanding the exact life history of *M. alternatus* is required. Since the late 1980s, the life cycle of *M. alternatus* has been accepted under the assumption that the final larvae pass four instars in the field. This study is revising the previous error for the life cycle hypothesis of *M. alternatus* by finding various instar pathways, which pathway is defined as the number of instars that larvae pass through prior to pupation. We confirm experimentally that the overwintered fourth or fifth instar larvae directly pupate to emerge as adults, indicating the presence of four and five instar pathways, respectively. The selection of instar pathway might be determined primarily by habitat temperature. This information will be useful to explain the variation of life history in *M. alternatus* populations worldwide based on the thermal environments, and also can be served to predict the northern distribution limit by applying the threshold degree-days for the completion of four instar pathway.

## Introduction

The Japanese pine sawyer, *Monochamus alternatus* Hope (Coleoptera: Cerambycidae), is a typical wood boring beetle, and this beetle transfers the pine wood nematode (PWN), *Bursaphelenchus xylophilus* (Steiner and Buhrer) that causes pine wilt disease (PWD)^1,2^. After making a wound in the bark surface, the females of *M. alternatus* lay eggs in the inner bark of pine tree branches^3,4^. Hatched larvae feed first on the phloem tissues of pine trees, and they enter deeply into sapwood tissues as they grow. Mature larvae make a pupal chamber in the wood for overwintering, and they are pupated in the chamber to emerge as adults next spring.

For a long time, the life cycle of *M. alternatus* has been accepted under the assumption that the larvae pass four instars in the field^4–8^. Overwintered, the third and fourth instars can emerge as adults in the current year without entering diapause^6^. But the first and second instar larvae after overwintering enter obligate diapause when reaching the fourth instar, and they emerge in the following year, showing a 2 year life cycle^6,9^. However, the hypothesis of this life cycle was subjected to serious challenge, because the fact that most *M. alternatus* larvae pass five instars was proved in the field experimentally in Jeju, Korea^10^.

The larval instar pathway in *M. alternatus*, which indicates the number of instars prior to pupation, may be a very important factor for understanding the population dynamics or potential distribution studies, because it basically determines the generation time. Belonging to the same Genus, *M. carolinensis* (Olivier) larvae showed one of several instar pathways in the laboratory: i.e. individual larvae were pupated after passing three, four, five, or six instars^11^. In *M. alternatus*, the instar pathway has not yet been exactly established, although it was partially known that the overwintered third and fourth instars^6^, or fourth and fifth instars^12^, could emerge as adults. This may be because it is difficult to examine the development of instars that are being processed secretly under bark in the field. Also, the repeat moulting of larvae to a maximum of 12th instar in the laboratory rearing, in spite of using natural food sources^10,13^, has become an obstacle to knowing the actual number of instars.

One approach to identify the number of instars in the field is to use the frequency distribution of head capsule width (HCW). However, this method may not be appropriate for *M. alternatus*, because the size of HCW is highly variable, and the frequency distributions among successive instars are largely overlapped^14,15^. So, this study applied a new method suggested by Go et al.^10^ that investigated the mandible exuviae of *M. alternatus* larvae in the feeding gallery: namely, the classes of mandible in size were investigated in the feeding gallery made by single larva. This mandible-based method for the discrimination of instar numbers has great merit, because during the larval feeding process, most mandibles are preserved without destruction^10^. Consequently, the objective of this study was to clarify the life cycle of *M. alternatus* with the confirmation of various instar pathways as well as the development of larval instars in response to change of environmental temperature.

## Results

The state of pupal chamber was different at the end of year (22 November in 2018) according to altitude (see Supplementary Table S2 online). All larvae at 200 m completed the pupal chamber, showing tightly plugged entrance of tunnel, and curved end of vertical tunnel. However, at 900 and 1100 m, some pupal chambers were incomplete. Also, all larvae showed diapause symptom at 200 m, while some of the larvae at 900 and 1100 m showed a milky white color that indicated no diapause symptom (see Supplementary Fig. S1 online).

The thermal environment of study sites was different in the accumulation of degree days based on a low threshold temperature of 12.4 °C (Table 1). Judging from the degree days, at 200 m, the larvae of *M. alternatlus* could complete the fifth instar until the end of 2018, namely, complete the development of fifth instar. But at 900 and 1100 m, the larvae did not reach the fifth instar: they developed to the late and middle states of the fourth instar, respectively.

**Table 1:** Number of *M. alternatus* adults according to the group and instar pathway. The degree days based on 12.4 °C were calculated, and converted to the age of larvae (the value in parenthesis) according to the thermal constant for each instar (see Supplementary Table S1 online). The term 5.C indicates that the larvae completed the development of 5th instar.

Altitude (m)	Degree days accumulated	First group		Second group		Third group		Instar pathway		
		n	Emerged	n	Emerged	n	Emerged	n	4 instar	5 instar
200	1110 (5.C)	9	8	11	10	11	7	25	18	7
900	695 (4.90)	9	5	11	7	11	4	16	15	1
1,100	632 (4.67)	9	5	11	7	11	2	14	14	0

**Figure 1:**
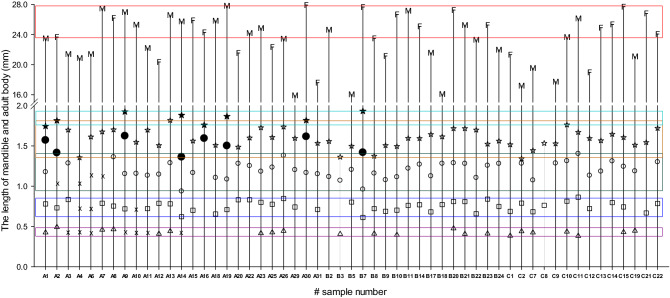
The number of larval instars indicated by the classes of mandible lengths in *M. alternatus*. The body length of the emerged adults was provided by the females (F) and males (M). The pine bolts infested with *M. alternatus* larvae were placed at different altitudes of A = 200 m, B = 900 m, and C = 1,100 m in the field. The sample number indicates the different groups: the first sample = (#1 to #9), the second sample = (#10 to #20), and the third sample = (#21 to #31) (please see the text for details). The first instar = △, the second instar = □, the third instar = ○, the fourth instar = ☆ in four instar pathway and ● in five instar pathway, and the fifth instar = ★. Thus, the symbols for final instars were ☆ and ★ in four instar and five instar pathway, respectively. The instars regarded as missing are indicated by the symbol “ × ”. The rectangular boxes on the graph indicate the range of each mandible length or adult body length.

Figure 1 shows the actual processes of instar development. The five instar pathway was largely dominant at 200 m compared with at 900 or 1100 m, showing 28.0, 6.3, and 0.0%, respectively. Although the thermal environment was sufficient for the completion of the fifth instar at 200 m, many larvae unexpectedly showed the four instar pathway. As expected by the accumulation of degree days at 900 and 1100 m, most larvae reached the fourth instar, and pupated with skipping the fifth instar (i.e., the four instar pathway was selected). Consequently, the instar pathway was significantly different by altitude, namely the thermal environment of habitat (χ^2^ = 6.91; *df* = 2; *P* = 0.0316).

The development of instars was highly variable in individual level, as seen in Fig. 1. But the length of mandible was significantly separated by each instar (Table 2, F = 1,035.39; *df* = 4, 172; *P* < 0.001). The range of mandible length in the first instar was clearly separated from that of the second instar. Also, the mandible range of the second instar was not overlapped at all with the third instar. Thus, missing mandibles in the first or second instars could easily be determined. The range of mandible length between the third and fourth instar or the fourth and fifth instar was somewhat overlapped.

**Table 2:** Mean mandible length (mm) of *M. alternatus* instars. The means with same letters in the column are not significantly different by Tukey test at *P* = 0.05 (*df* = 4, 172; *F* = 1035.39, *P* < 0.001). Not significant (^ns^) by a two sample *t* test between male and female (The 1st instar, *df* = 19; *t* = 0.03; *P* = 0.9760, the 2nd instar, *df* = 42; *t* = 0.89; *P* = 0.3775; the 3rd instar, *df* = 44; *t* = 0.11; *P* = 0.9120, the 4th instar, *df* = 51; *t* = 1.14; *P* = 0.2611; the 5th instar, *df* = 6; *t* = 0.44; *P* = 0.6753).

Instar	n	Mandible length (mean ± SEM)			Range	
		All combined	Male	Female	Minimum	Maximum
First	22	0.428 ± 0.0060e	0.429 ± 0.0114^ ns^	0.429 ± 0.0065	0.382	0.492
Second	45	0.751 ± 0.0092d	0.758 ± 0.0134^ ns^	0.741 ± 0.0130	0.611	0.863
Third	47	1.200 ± 0.0146c	1.204 ± 0.0199^ ns^	1.201 ± 0.0222	0.937	1.404
Fourth	55	1.574 ± 0.0152b	1.592 ± 0.0219^ ns^	1.558 ± 0.0190	1.342	1.818
Fifth	8	1.843 ± 0.0248a	1.854 ± 0.0384^ ns^	1.831 ± 0.0363	1.745	1.932

The growth rate of mandible length between two successive instars was significantly different according to instars (Table 3, F = 131.0; *df* = 3, 109; *P* < 0.001), and it decreased significantly between later instars; resulting in no difference between the growth rates of the third to fourth instar and the fourth to fifth instar. An extreme case for the growth rate was 1.05 between the third and fourth instar in #C2, showing nearly no growth during the development.

**Table 3:** The relative ratios (growth rate) of the (*i* + *1*)th/*i*th mandibles of *M. alternatus* instars. The means with same letters in the column are not significantly different by Tukey test at *P* = 0.05 (*df* = 3; 109; *F* = 131.0; *P* < 0.001).

Growth rate (*i* + *1*)th/*i*th mandible)	n	Mean ± SEM	Range	
			Minimum	Maximum
2nd/1st	20	1.82 ± 0.038a	1.49	2.26
3rd/2nd	40	1.60 ± 0.011b	1.46	1.81
4th/3rd	46	1.32 ± 0.014c	1.05	1.54
5th/4th	7	1.22 ± 0.045c	1.10	1.38

The length of adult body was highly variable, ranging from a minimum 15.93 mm (#A29) to maximum 27.95 mm (#A30) (Fig. 1). The adult length was not significantly different between males and females in all cases (see Supplementary Table S3 online), although females showed a larger trend than males. When males and females were combined, the adult body length was significantly larger in five instars pathway than in four instars pathway at 200 m (two sample *t* test: *t* = 2.14; *df* = 23; *P* = 0.0432).

**Figure 2:**
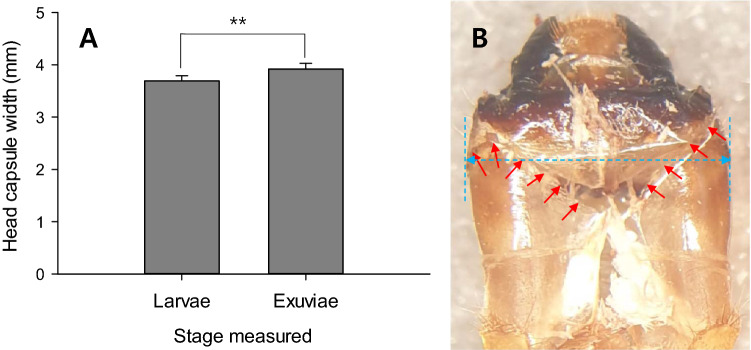
Comparison of the head capsule width (HCW) of *M. alternatus* larvae measured at different state. The vertical bar on graph indicates s.e.m. A: The difference of HCW between living larvae and the exuviae, B: Dorsal view of larval exuviae with ecdysial line split (indicated by arrows). The dotted line is the measurement line of HCW. The ** symbol means significantly different by paired *t* test at *P* = 0.01.

**Figure 3:**
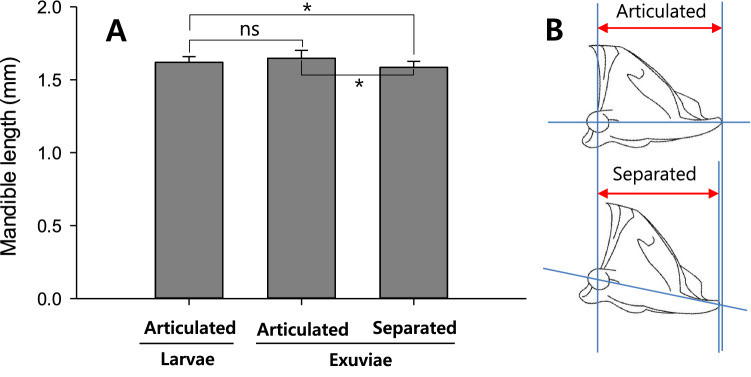
Comparison of the mandible length of *M. alternatus* larvae measured at different state. The vertical bar on graph indicates s.e.m. A: Difference of mandible length measured on living larvae (namely articulated with the head capsule of larvae) and the state articulated or separated from the head capsule of exuviae; B: Change of measurement line according to the state of mandible that is articulated with or separated from the larval head capsule. The * symbol means significantly different by paired *t* test at *P* = 0.05.

The samples of the first group, which was examined on 22 November 2018, provided comparative data for the head capsule width (HCW) and mandible length: the size differences before and after overwintering or molting. The final HCW measured on living larvae in 2018 was significantly smaller than those measured on the exuviae left after pupating in 2019 (Fig. 2A: *df* = 18, *t* = 8.12, *P* < 0.0001). But the difference was not large, showing just 0.228 mm. The mandible length of final instars was significantly shorter when measured on separated state from living larvae or exuviae (Fig. 3A: living larvae, *t* = 2.23; *df* = 15; *P* = 0.0417, exuviae, *t* = 5.05; *df* = 15; *P* = 0.0002). However, there was no significantly difference between the mandible length measured (articulated state) on living larvae and the exuviae (*t* = 0.99; *df* = 15; *P* = 0.3372).

**Figure 4:**
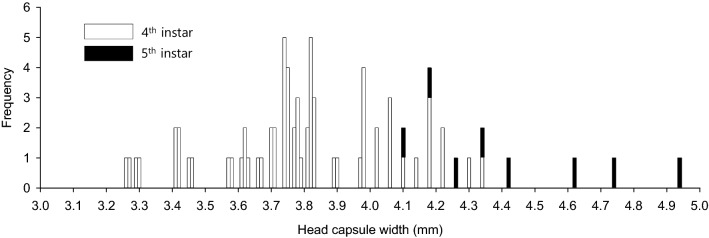
Frequency distribution of head capsule width of *M. alternatus* larvae in the final instars, which is based on HCWs measured on the exuviae found in the pupal chamber. The frequencies of the fourth and fifth instars are denoted by open and solid bar, respectively (n = 51). This distribution was made using a class interval of 0.04 mm.

The frequency distribution of HCW of final instars (namely found in pupal chamber) was widely spread, and showed multi-modality with several peaks of 3 or 4 (Fig. 4). The right tail consisted mostly of the fifth instars. The single group of fourth instars produced all the heterogenous minor peaks.

## Discussion

The approach for determining the number of instars in insect species has frequently applied the frequency distribution of head capsule width^16–18^. But this method can mislead actual instar numbers, since the HCWs of successive instars largely overlap in the field^18^. Our novel approach for the determination of instar number is basically different from previous methods using frequency distribution of HCWs. Our method can exactly determine instar numbers without confusion by overlap error in HCWs, because it counts directly the number of mandible classes in the same feeding gallery. To the degree to which our approach reflects the real circumstances in the field, the present results will provide actual biology to understand the life history of *M. alternatus*.

The HCWs of *M. alternatus* larvae that were measured on the exuviae in the pupal chambers were significantly longer than those measured when the larvae were alive in the previous year (see paired *t* test). The difference was just average 0.228 mm, which did not indicate another molting, because the difference was more than 0.9 mm between instars in late larvae^10,14^. In our experimental condition, the larvae could not develop to the next instar in the spring, since the nutritional inner barks of pine bolts were all removed, and only wood tissues inside that were nutritionally invalid were available. Thus, it might be caused by other factors, such as head capsule widening after the last molting. Actually, the ecdysial line was split after molting, which existed on the dorsal side of the head capsule (Fig. 2B).

Also, the mandible length was statistically different between the articulated and separated state from the head capsule. This difference can be explained by the geometric structure of the mandible. When the mandibles are articulated to the larval head capsule, the measurement line of mandible length and the straight line from the center of condyle to the tip of mandibular incisor are on almost the same horizontal plane (Fig. 3B). But when separated, the mandible is slightly inclined towards the tip of incisor. This shortens the measurement line, because the straight line from the center of condyle to the tip of incisor is slightly declined. Consequently, the larvae that entered overwintering in 2018 must be pupated without further molting in the next spring, 2019.

The variation in the adult size of *M. alternatus* is known to be very high, ranging from 17 to 28 mm in body length^4,12^. In this study, the adult body length was significantly different between instar pathways. Also, Guo et al.^12^ reported that the heavier overwintered fifth instar produced larger adults than the lighter fourth instar in China. Female adults were significantly longer than males in many studies^12^, while there was no statistically difference between sexes in our study, although in most cases, females showed a larger trend. In any case, it seems to be true that the males classified by the mandible length and body length of adults are frequently in the low quartile (see Supplementary Fig. S2 online). The frequency distribution of the head capsule width of final instars in this study showed multi-modality. Also, very small males were located at the left tails.

Pershing and Linit^11^ reported that *M. carolinensis* developed through three, four, five, or six instars prior to pupation. Their conclusion was based on the number of instars of larvae reared on artificial diet. In our study, three or six instar pathways were not found. The minor peak located on the left side of the HCW distribution was formed mostly by the fourth instars (Fig. 4). Also, the right peak was extremely contributed by the fifth instars. The sample number of #C2 (Fig. 1) showed very interesting instar development. When judged by the mandible size, the third instar developed to the fourth instar without meaningful size extension. Further studies are required to conclude whether the third instar would be able to pupate directly, which has been unknown still since reports by Togashi^6^. In China, Guo et al.^12^ reported that the fourth and fifth instars entered overwintering and emerged directly to adults in the next year, although they applied only the HCW for the determination of instars. That report is consistent with our results.

In the present study, it was experimentally confirmed that the fourth or fifth instars of *M. alternatus* larvae were directly pupated, indicating the presence of four and five instar pathways. The five instar pathway was selected more in warm condition (200 m) than in cool condition of (900 or 1,100) m, where most larvae passed four instars. The accumulated degree days must be an essential influencing factor for the selection of instar pathways, because almost all larvae selected the four instar pathway in the conditions of insufficient degree days for the development up to the fifth instar. At 200 m, 62% of larvae stopped the development during the fourth instar by selecting the four instar pathway, although the accumulated degree days were sufficient for the completion of the fifth instar. This higher four instar pathway than expected might be caused by the innate biological property and diapause ecology of *M. alternatus*. As described in Supplementary Information II (online), all larvae at 200 m were found with penetration holes plugged by wood debris, which were examined on September 21, 2018. Unfortunately, we have not been able to confirm the diapause symptoms of the larvae inside the pine bolts and the completion of the pupa chamber. But the state of penetration holes indicated that many larvae probably stopped the development before reaching the fifth instar by unknown cues of diapause induction much earlier than late September. In this case, thus, it is thought that the instar pathway of *M. alternatus* is controlled by another factor such as their own innate diapause ecology in addition to temperature.

Previously, we reported five instars of *M. alternatus* larvae in Jeju, Korea^10^. The experiments were designed to obtain the larvae with full growth by inoculating hatched larvae in very early season, late May 2017, and also the samples of pine bolts were treated at coastal warm area. If an innate factor had operated, any four instar pathway would have been found. We believe that the individual #5 was through four instar pathway (see Fig. S3 of the SI in Go et al.^10^), in which the mandible of the fourth instar in the feeding gallery must be misjudged as missing, because the authors did not notice a four instar pathway at that time. Also, the previous results strongly showed five instar pathway, compared with the present results at 200 m. This might be because the hatched larvae were inoculated much earlier (late May) in the previous study, than in the present study (mid-July). In other words, most larvae were able to complete the five instars before a cue of diapause induction.

As a tentative conclusion, the selection of instar pathway in *M. alternatus* is likely determined by the interaction of the innate biological property and habitat temperature environment, in which the factor of habitat temperature (degree days) basically regulates the selection. Under such assumption, the four instar pathway that has been accepted for a long time in Japan is understood. The hypothesis of 1 or 2 year life cycle of *M. alternatus* with four instars has been established on the basis of field experiments conducted in Ishikawa province, Japan^6^, where air temperatures were somewhat lower than at Jeju (see geographical positions in Supplementary Fig. S3, online). As seen in Supplementary Table S6 (online), accumulated degree days were much lower in Ishikawa (Japan) than in the study sites for larval development of *M. alternatus* in China or Korea, where the five instar pathway was recorded. So, it seems to be conclusive that *M. alternatus* passed four larval instars in the northern areas of Japan. Because Korean strain of *M. alternatus* is regarded as the same as the Japanese strain (*M. alternatus endai* Makihara^19^), the difference in larval pathway between two countries must be caused by the temperature difference of habitat. Additionally, the individuals of *M. alternatus* in 2 year life cycle may not contribute largely to the population build up, because of high mortality caused by the invasion of various secondary wood-boring beetles (see Supplementary Table S5).

In the growth rule of insect larvae, Slansky and Scriber^20^ have established a theory that ‘larval critical weight is the minimal weight for pupation that allows for the production of a functional adult’. Therefore, the critical stage is completed by a relatively constant size^21,22^. This theory has been developed into a hypothesis for the number of instars by Esperk et al.^23^, who suggested the concept of ‘compensation scenario’ to explain the plasticity in the instar number of insects. They have established an adaptive scenario that ‘instar number increases in adverse conditions when larvae fail to reach a species-specific threshold size for metamorphosis’. However, these theories may not be consistent with our results, since *M. alternatus* selected the four instar pathway in adverse cool environment. A few insect species showed exceptional responses, in which instar number was higher in favorable conditions (reviewed in Esperk et al.^23^): *M. carolinensis* (Coleoptera: Cerambycidae) in favorable temperature at 25 °C than at 20°C^11^, *Psacothea hilaris* (Coleoptera: Cerambycidae) in food *ad libitum*^24^, *Diploptera punctata* (Dictyoptera: Blaberidae) in low density^25^, *Chorthippus brunneus* (Orthoptera: Acrididae) in low density and favorable temperature^26^, and *Melanoplus differentialis* (Orthoptera: Acrididae) in low density^27^.

In the population growth of *M. alternatus*, the selection of five instar pathway has highly advantageous merit, because larger females emerged from the fifth instar larvae^12^ that can lay more eggs^28^ of larger size^29^ than smaller females from the fourth instars. Thus, *M. alternatus* will select the five instar pathway to increase the reproductive ability in a favorable environment. Also, in an adverse cool environment, this beetle may be able to establish the population by selecting the four instar pathway. Such ability can be another trait to increase their fitness in variable environments. That is, the biological properties of *M. alternatus* that can select the four instar pathway may extend the distribution limit toward higher altitude, as far as the area where the accumulated degree days can reach the fourth instar; because this species is known to have good tolerance against chilling^30^. Consequently, it is seems that the selection strategy of instar pathway is an adaptive strategy of *M. alternatus* to variable thermal environments, rather than the theory of larval critical weight^20^ and compensation scenario^23^; thus this beetle can increase the fitness against unpredictable changes of thermal environment. In insect species, also, there seems to be a mechanism to increase the number of instars to produce good offspring in a favorable condition, namely instar number adjusted to a maximizing reproduction. Of course, this hypothesis requires more verification in the future.

In the present study, we have provided a new direction for understanding the life cycle of *M. alternatus*: namely the selection of instar pathway, and its regulation mechanism. This information will be useful to explain the variation of life history in *M. alternatus* populations worldwide based on the thermal environments as seen in Supplementary Table S6 and Fig. S3 (online).

## Methods

### The procedure for egg collection

Adults of *M. alternatus* were obtained from the branches of pine trees infested with the larvae in Jeju city, Korea, which were collected during February in 2018. The preparation of eggs from emerged *M. alternatus* was conducted by the protocol of Go et al.^10^ (see Supplementary I online).

### Inoculation of hatched larvae under the barks of pine bolts

Collected eggs of *M. alternatus* were handled and were introduced into pine bolts using the method of Go et al.^10^ modified from Togashi^31^ (see Supplementary I online).

### Investigation of the development of larval instars in the field

The pine bolts inoculated with the first instar were placed at three altitudinal sites of 200, 900 and 1,100 m on Mt. Hallasan, Jeju (17 July, 2018). This strategy was to provide different thermal environment for larval development, and obtain different stages of larval instars when they reached overwintering state. At each site, 33 pine bolts were treated in field cages of (120 cm × 70 cm × 60 cm) covered with a fine wire mesh (3 mm). The temperatures in the cages were also monitored using a data logger (HOBO MX2301, Onset Computer Corporation, MA, USA), for use later to calculate degree days (namely, the physiological age of larvae, see Supplementary Table S1 online).

The samples of pine bolts were divided into three groups, according to the investigation procedure at each site.

#### The first group collected 22 November, 2018

Nine pine bolts were collected at each altitudinal site on 22 November, 2018, to investigate the larval state that had entered into overwintering, and if there was the development of instars after overwintering in the next spring. At 200 m, the pine bolts were preliminarily examined on 21 September, to check whether the larvae made a pupal chamber (see Supplementary Table S2 online).

The outer bark and feces with wood shreds were clearly removed from the collected pine bolts, and kept in a plastic bag (25 cm × 33 cm), to use later for the investigation of mandibles (see below for details). Then, the pine bolts were split using a hand axe to examine the larvae inside.

The head capsule width (HCW) of alive larvae was measured across the greatest width of the head capsule, using a digital microscope (Dino-Lite, AD4113TL-MA1(R4), ANMO Electronics Co.), as the method suggested by Go et al.^10^. The mandibles of *M. alternatus* were articulated with a thickened part of the head capsule in the region of the clypeus. Thus, the length of mandible of the condyle center to incisor cusp was measured in the state where mandibles were articulated with the head capsule, according to the recommendation of Go et al.^10^. Also, larval weight, the diapause symptom of larvae, and the structure of pupal chamber were checked to compare the larval state among altitudinal sites (see Supplementary Table S2 online). The dead larvae were excluded from the measuring.

After examining the larvae, the split pine bolts were fitted with putting the larvae back inside, and wrapped using a stretch film (polyethylene chloride film, CLEANWRAP, Seoul). These pine bolts were placed in a field cage (50 cm × 60 cm × 40 cm) covered with a fine wire mesh (3 mm), installed in an experimental farm at Jeju National University. Also, temperature in the field cage was monitored using the same type of HOBO logger above.

On 18 March 2019 after sufficient days for diapause termination (> 90 days), the pine bolts were moved, and placed individually in a plastic bottle (diameter 15.6 cm, height 20 cm) in insect rearing room at (25.0 ± 1.0) °C, a photoperiod of 16:8 (L:D) h, and RH > 40%). Wet cotton pads were placed inside the cages to prevent the desiccation of pine bolts. After the emergence of adults, which was checked for 2–3 times a week, the head capsule width (HCW) and mandible length (articulated state) were measured on the larval exuviae in pupal chambers, using the same method above that was applied to live larvae. Also, the mandible length was measured on the state separated from the head capsule of larval exuviae. The body length of adults was measured as the distance from the end of vertex to the tip of elytral apex. Female and male adults were distinguished by the white band on the antennae of females^4^, and comb-like trichome on the end of female abdomen^32^.

When single exuviae was found in each pine bolt (namely in pupal chamber), it was assumed that the larval instar on 22 November of 2018 was directly pupated without further molting in 2019, because the pine bark and feces with wood shreds were clearly removed previously in 2018. Also, throughout all the experimental procedures, including the other experiments below, the larva and adult from the same pine bolt were traced by the same reference numbers, to examine the change of morphometric variables in each developmental stage.

The number of mandible exuviae of *M. alternatus* larvae were examined in the feeding gallery of pine bolts prepared previously above. The barks were carefully examined to separate all the attached feeding residues. The total feeding residue of each pine bolt was soaked in tap water in plastic container (300 ml) (approximately 1:2 of feeding residue:water), to loosen the lumps of feces and wood shreds. At a time, one teaspoon of feeding residue was transferred to a plastic petri-dish (diameter 90 mm, depth 15 mm) to find out mandibles under a stereomicroscope of (8–20) × . The found mandible length was measured using the same method above.

The number of mandible classes (strata) in size was used to determine the number of instars passing in the pine bolts^10^. That is, three and four strata of mandibles in the feeding gallery were regarded as the pathway of four and five instars, respectively, because the final instars were located in the pupal chamber. It was not easy to find all the successive strata of mandible in a pine bolt, since during the feeding process of *M. alternatus* larvae, some mandibles were probably destroyed and lost. Therefore, a missing instar was determined by the ranges of mandible length in each instar, or the relative size of mandibles calculated between two successive mandibles of instars found in a specific pine bolt.

#### The second group collected 15 May, 2019

Eleven pine bolts each at (200, 900, and 1,100) m were collected on 15 May 2019, and they were placed individually in plastic cages in the insect rearing room using the same method above. After adult emergence (daily observation was made), the mandibles in feeding residues and larval exuviae in pupal chamber were investigated using the same methods above.

#### The third group kept in the field

The remaining pine bolts (namely each ten bolts at 200, 900, and 1100 m) were kept in the original sites, until adult emergence. Previously on 20 May 2019, the pine bolts were individually placed in a wire-mesh sleeve bag (1 mm mesh size). When the emergence of adults was made (checked 2–3 times per week), the wire-mesh sleeve bags, including pine bolt and adult, were brought to the laboratory; and they were investigated using the same method above.

### Data analysis

All data sets were tested for the normality before statistical analysis. The difference of HCW between living larvae and larval exuviae (final instar) was examined by paired *t* test. Also, paired *t* test was applied to compare the mandible length between articulated to the larvae (or exuviae) and separated. The mandible length of instars was analyzed by one-way GLM (generalized linear model^33^), and the means were separated by Tukey’s range test. The difference of mandible length between males and females was analyzed by *t* test. The relative ratios (defined as growth rate) of the (*i* + *1*)th/*i*th mandibles of *M. alternatus* instars were calculated and analyzed by Tukey’s range test after one-way GLM.

In this study, the term of instar pathway was defined as the number of instars that each larvae passed through prior to pupation^11^. The frequency of adults according to the two factors of instar pathway and altitude was tested by χ^2^–test. The effects of instar pathway and sex (or altitude and sex) on the length of adult body were analyzed by two-way GLM^33^. Means were separated by Tukey’s range test at *P* = 0.05, or two sample *t* test (see Supplementary Table S3 online).

## Supplementary information

Supplementary Information.
