# Supplementary material for: A new direction to understand the life cycle of the Japanese pine sawyer considering the selection strategy of instar pathways
Source: Sci Rep. 2020 Oct 12;10:16952. doi: 10.1038/s41598-020-73344-1 (PMC7552422; doi:10.1038/s41598-020-73344-1)
Supplement: Supplementary file 1 — Supplementary Information. [file 41598_2020_73344_MOESM1_ESM.pdf]

Supplementary Information

A new direction to understand the life cycle of the Japanese pine sawyer considering the selection strategy of instar pathways

Su Bin Kim and Dong-Soon Kim

Supplementary Information I. Supplementing the experimental methods

The procedure for egg collection

Adults of *M. alternatus* were obtained from the branches of pine trees infested with the larvae in Jeju city, Korea. The branches were collected during February of 2018, cut into 1 m long logs (90 pine logs), then placed in a field cage of (6.5 m × 4.8 m × 3.0 m) covered with fine wire mesh (3 mm) installed at Mt. Halla-san, Jeju city. The adults that emerged in the field cage were introduced into an acrylic rearing cage (110 cm × 70 cm × 55 cm) in the laboratory (25.0 ± 1.0) °C, a photoperiod of 16:8 (L:D) h, and RH > 40 %). Pine shoots (of 20 cm length) from new growth or 1 yr old twigs were used as the food supply of *M. alternatus* adults, by providing two to five shoots every (2–3) d, depending on the consumption.

The oviposition substrates were prepared by the protocol of Go et al. (2019). That is, *P. thunbergii* trees (5–6 yr old with a diameter of (5–8) cm) were collected from Jeju city, Korea, then 15 to 20 cm long bolts were made. After leaving for 1 d at room temperature to prevent the secretion of resin, these pine bolts were submersed in water for (3–5) d. Pine bolts were introduced into the rearing cage after drying in the shade for 1 d. Pine bolts were replaced 3 times a week in the rearing cage, and the collected pine bolts were held in a refrigerator (4 °C), until used for various experiments, as well as this study. In the rearing cage, a minimum of fifteen adult pairs were kept during egg collection.

Monochamus alternatus eggs were collected from oviposition scars on pine bolts by the following method of Go et al. (2019). Pine barks were cut out to 40 mm length and 20 mm width at the center of oviposition scar. The eggs were collected in the space between phloem and phellogen about 7 mm above oviposition scars.

Inoculation of hatched larvae under the barks of pine bolts. Collected eggs of *M. alternatus* were hatched on filter paper placed in a small petri-dish (diameter 5 cm, depth of 1 cm) held at 25 °C (RH > 60 %, 16:8 (L:D) h), using the method of Go et al. (2019). The small petri-dish was also placed in a larger petri-dish (diameter 10 cm, depth 4 cm) lined with moistened cotton on the bottom. A group of 20–30 eggs was placed in one small petri-dish, preparing more than a total of 120 eggs for this experiment.

The hatched larvae between 9 and 13 July in 2018 were introduced into pine bolts using the following method of Go et al. (2019) modified from Togashi (2014). Pine bolts of 15–20 cm length and 5 cm diameter were previously prepared, as described earlier. Cut ends of these bolts were sealed with vinyl tape to prevent drying. To make a space for the inoculation of *M. alternatus* larvae, a small square of bark (10 mm × 10 mm) was removed from pine bolts at the mid-point. Then a pit was made on the exposed xylem with a medical knife. Larvae were introduced singly into the pit, covered again with the bark square, and fastened with a rubber band.

References (Suppl. I)

Go, M. S., Kwon, S. h., Kim, S. B., Kim, D.-S., 2019. The Developmental characteristics for the head capsule width of *Monochamus alternatus* (Coleoptera: Cerambycidae) larvae and determination of the number of instars. J. Ins. Sci. 26, 1-9. (doi: 10.1093/jisesa/iez010)

Togashi, K., 2014. Effects of larval food shortage on diapause induction and adult traits in Taiwanese, *Monochamus alternatus alternatus*. Entomologia Experimentalis et Applicata 151, 34-42.

Supplementary Information II. Additional data and the analysis

1. Thermal constant of larval instars

The time required to complete the development of each larval instar of *M. alternatus* was standardized using degree days based on a common low threshold temperature. The average low threshold temperature of 12.4 °C for the development time from overwintering larvae to the completion of 5th instar (Kwon, 2017) was regarded as the common low threshold temperature. Then degree days (DD) for the development completion of each instar (namely thermal constant *K*) were calculated by the following equation (Lin et al., 1954):

$$K = d_i\,(T_i - T_b)$$

where, T_i is the temperature of incubation, d_i is the mean number of days in incubation at the *i*-th temperature, and T_b is the developmental threshold, namely 12.4 °C in this case.

Finally, the thermal constants were calculated to (93.4, 150.1, 200.2, 280.5, and 312.6) DD for the (1st, 2nd, 3rd, 4th, and 5th) instar, respectively. Also, cumulative degree days become (243.6, 443.8, 724.3 and 1,036.9) DD for the completion until the 2nd, 3rd, 4th, and 5th instar, respectively.

The criterion of thermal constants was used to predict the development state (physiological age) of larval instar on a specific date in the field. In this regard, for example, 695 DD indicates 4.90 instar, because of (443.8 DD = 4.0 instar) and (251.2/280.5 = 0.90). When degree days were over 1,036.9 DD, it was termed “5.C”, because the development of the 5th instar was completed.

Supplementary Table S1. Degree days (DD) for the development completion of each instar (namely thermal constant *K*) of *M. alternatus* based on low threshold temperature of 12.4 °C.

Temperature (°C)	1st instar		2nd instar		3rd instar		4th instar		5th instar	
	d_i	*K*	d_i	*K*	d_i	*K*	d_i	*K*	d_i	*K*
18	16.2	90.7	25.9	145.0	33.7	188.7	46.8	262.1	48.2	269.9
22	9.7	93.1	15.6	149.8	22.4	215.0	30.2	289.9	33.7	323.5
26	7.1	96.6	12	163.2	15.1	205.4	21.6	293.8	24.1	327.8
30	5.3	93.3	8.1	142.6	10.9	191.8	15.7	276.3	18.7	329.1
34[1]	5.1		7.7		10.9		16		18.1	
Mean of *K*		93.4		150.1		200.2		280.5		312.6
Cumulative				243.6		443.8		724.3		1,036.9

[1]Data points at 34 °C were excluded, because they were out of linear relationship.

2. The structure of pupal chamber and the state of larvae in the end of 2018

The mature larvae of *M. alternatus* excavate a tunnel (pupal chamber) in the wood before overwintering. The larvae first dig a horizontal hole of about 2 cm depth into the wood surface, and then make a vertical tunnel along the direction of the wood fiber; finally, the vertical tunnel is curved toward the wood surface at the end (Togashi, 1989b). Also when they enter diapause, the larvae tightly plug the entrance of the tunnel (penetration hole) with wooden fibrous shreds. To make a preliminary examination of whether the larvae made a pupal chamber, nine pine bolts at 200 m were examined on 21 September 2018. The presence of entrance plug of tunnel was examined on the surface, without splitting the pine bolts.

On 22 November 2018, the structure of the pupal chamber and state of larvae in the pine bolts were investigated in each of nine pine bolts placed at (200, 900, and 1,100) m. The pine bolts were split using a hand axe to examine the larvae inside and tunnel (pupal chamber). The larvae in tunnel were classified by diapause symptom, based on the following criteria recommended by Kimura (1974) and Togashi (1991): body is yellowish white, whitish yellow, or yellow color; no food in the gut. Furthermore, the pupal chamber was checked by the structure (Togashi, 1989b): the plug of tunnel entrance (no plug, or tightly or loosely plugged), and the vertical tunnel (without or with the curved end). The curved end of the vertical tunnel indicates a completed pupal chamber, since the exit hole of adults is made through it.

All larvae at 200 m were observed with penetration holes plugged by wood debris on 21 September, 2018. Also, they had completed the pupal chamber by the end of 2018, and showed diapause symptom (Table S2 and Fig. S1). But some larvae at (900 or 1,100) m were found with unclear diapause symptom showing milky white color and not curved tunnel, although no larvae had food in the gut.

Supplementary Table S2. The number of larvae according to the structure of pupal chamber and diapause symptom examined on 22 November, 2018

Altitude (m)	n	Sur-vived	The structure of pupal chamber				The diapause symptom of larvae			
			Plug of entrance		End of tunnel		Body color			No food in the gut
			Tight	Loose	Curved	Not curved	Yellow	Whitish yellow	Milky white	
200	9	8	8	0	8	0	4	4	0	8
900	9	6	4	2	5	1	0	4	2	6
1,100	9	7	3	4	4	3	1	2	4	7

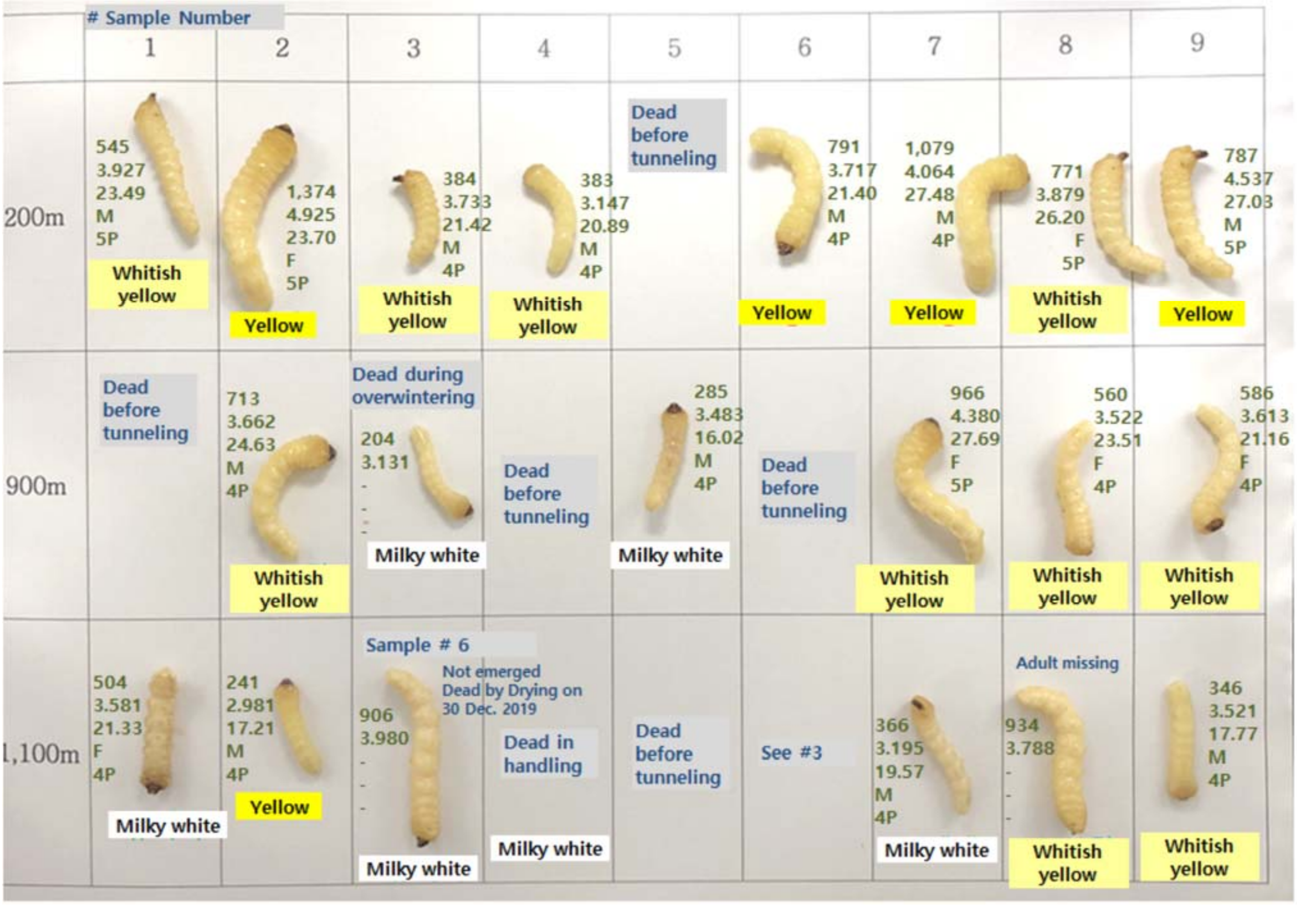

Supplementary Figure S1. The appearance of larvae examined on 22 November 2018. The larval weight (mg), HCW (mm), the body length of adults (mm), sex (M = male, F = female), and instar pathway (4P = four instar, 5P = five instar) were provided by each larva.

3. The body length of adults according to altitude and sexes

The results at 200 m provided the data sets classified by two factors of instar pathway and sex (Table S3). The effects of instar pathway and sex on the length of adult body were analyzed by two-way GLM (generalized linear model; SAS Institute, 2000). The effects of instar pathway, sex, and the interaction of two factors were not significantly different on the adult length at $P = 0.05$ (Instar pathway: $F = 4.28$; df = 1, 21; $P = 0.0510$, Sex: $F = 0.51$; df = 1, 21; P: = 0.4839, the interaction: $F = 0.01$; df = 1, 21; $P = 0.9266$). Since the effect of pathway has considerable F-value with nearly significant probability, the males and females in the same instar pathway were combined to apply two-sample t-test. When combined, the adult body length was significantly larger in the 5 instar pathway, than in the 4 instar pathway ($t = 2.14$; df = 23; $P = 0.0432$).

In the 4 instar pathway (the analysis of two-way GLM), the effects of altitude and sex on the length of the adult body were not significant at $P = 0.05$ (Altitude: F = 0.15; df = 2; $P = 0.8627$, Sex, $F = 3.14$; df = 1; $P = 0.0842$, the interaction of altitude and sex: $F = 2.16$; df = 2; $P = 0.1286$). Since the effect of interaction between altitude and sex has considerable F-value with low probability, the data sets were separated to examine the difference between males and females on adult size. The results of two-sample t-test showed no significant difference in adult size between males and females (200 m, $t = 0.69$; $P = 0.4991$, 900 m, $t = 1.64$; $P = 0.1285$, 1,100 m, $t = 1.80$; $P = 0.0993$).

Supplementary Table S3. The length of adult body by instar pathway, sex, and altitude.

Pathway	Sexes	Altitude (m)		
		200	900	1,100
4 instar pathway	Female	(21.57 ± 1.351) ns[1] (6)	(24.84 ± 0.914) ns (6)	(24.18 ± 1.159) ns (6)
	Male	(23.30 ± 0.917) (12)	(22.03 ± 1.441) (8)	(20.92 ± 1.421) (7)
	Combined	(22.32 ± 0.863)*[2]	(23.24 ± 0.962)	(22.68 ± 0.985)
5 instar pathway	Female	(25.33 ± 1.327) (4)	27.69 (1)	-[3]
	Male	(26.04 ± 0.953) (3)	-	-
	Combined	(25.57 ± 0.513)	-	-

The values in parenthesis indicate sample size.

[1] Not significant at P = 0.05 by two sample t-test between females and males (200 m, t = 0.69; P = 0.4991, 900 m, t = 1.64; P = 0.1285, 1,100 m, t = 1.80; P = 0.0993).

[2] Two-sample t-test between 4 and 5 instar pathways in combined data of females and males at 200 m (t = 2.14; df = 23; P = 0.0432).

[3] No available data.

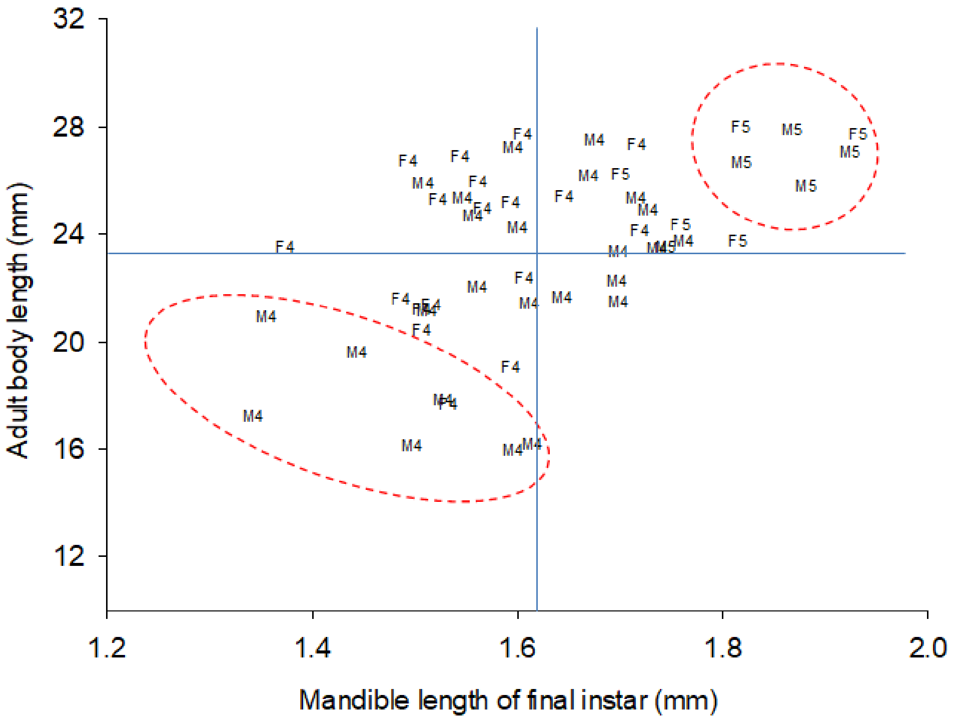

Supplementary Figure S2. Two-dimensional arrangement of adult body length according to the mandible length of final instar. The symbols indicate M = male, F = female, 4 = 4 instar pathway, and 5 = 5 instar pathway. The vertical and horizontal lines indicate each average value.

4. Emergence and survivorship of *M. alternatus* in the study pine bolts

In this experiment, 57.0 % of *M. alternatus* emerged as adults in the following year. The emergence rate (39.4 %) of the field group (the third group) was lower compared to the groups (avg. 68.2 %) that were held in the laboratory (Suppl. I Table S4). Until the end of 2019 (the 30th December), two larvae were alive in pine bolts. But the larvae were not active enough to survive (Suppl. I Table S5).

Most deaths occurred during larval stage living in the feeding gallery, which accounts for 75.7 % of mortality. Among mortality factors, death by invasion of secondary wood-boring beetles was observed (Suppl. I Table S5), although the pine bolts were put into mesh sleeves to prevent such invasion (namely accidental contamination).

Monochamus alternatus is a typical primary that attacks and kills healthy trees, while various secondary wood-boring beetles attack only dying or dead trees (James and Shaw, 2010; Rassati et al., 2016). Because of the competition for food resources, the secondary wood-boring beetles can seriously affect the survival of primary wood-boring species. In particular, pupal or quiescence larval stage in pupal chamber that lack defense ability may be very vulnerable to the invasion of secondary wood-boring beetles. In the field condition, secondary wood-boring beetles frequently attack the pine trees killed by *M. alternatus* (Jeong et al., 2010). Thus, individuals of *M. alternatu*s with 2 yr life cycle that were reported by Togashi (1989b) may not contribute largely to the population build-up, because of such high mortality caused by various mortality factors, including secondary wood-boring species. In this study, if the mesh sleeves for pine bolts had not been set, then most pine bolts would have been infested with secondary wood-boring beetles.

Supplementary Table S4. The number of emerged or dead *M. alternatus* according to the experimental groups.

Group	Altitude (m)	n	Emerged	Dead	Alive (larvae)
**First**	200	9	8	1	
	900	9	6	4	
	1,100	9	5	3	1
	**Subtotal**	**27**	**19**	**8**	**1**
**Second**	200	11	10	1	
	900	11	6	5	
	1,100	11	7	4	
	**Subtotal**	**33**	**23**	**10**	**0**
**Third**	200	11	7	4	
	900	11	4	7	
	1,100	11	2	8	1
	**Subtotal**	**33**	**13**	**19**	**1**
**Total**		**93**	**55**	**37**	**2**

The third group is field-aged individuals up to adult emergence.

Supplementary Table S5. Partial life table of *M. alternatus* in our study population

Category	Stage	Position found	No.	Remark or mortality factors
**Total**			**93**	
Dead	Larvae	Feeding gallery	28	
		Pupal chamber	5	Each one case:<br>Infested by secondary wood-boring beetles
	Pupae	Pupal chamber	2	
	Adults	Pupal chamber	2	Failed to escape
	**Subtotal**		**37**	
Alive	Larva 1	Pupal chamber	1	Severely shrunk by drying
	Larva 2	Pupal chamber	1	Abnormal vitality
**Emerged to adults**			**55**	

References (Suppl. II)

James, E. R., Shaw, D. C., 2010. Common insect pests and diseases of shore pine on the Oregon coast (*Pinus contorta* Douglas ex. Louden var. *contorta*). Oregon State University, EM 9008, 14 pp.

Jeong, C. S., Yiem, J. O., Go, S. H., Moon, I. S., Han, H. L, Lee, S. H., Jeong, Y. J., Shin, S. C., 2010. A field guide for the identification of vector species that transfer pine wilt disease. Administrative publication No. 11-1400377-000380-01, Kora Forest Research Institute. (In Korean)

Kimura, S., 1974. Development and growth of the pine sawer in relation to temperature. I. The influence of low temperature on the larval period of pupation, pp. 141-144. *In* Proceedings, Trans. 26th Annu. Meet., Tohoku Branch Jpn. For. Soc., Japan.

Kwon, S. H., 2017. A population model of *Monochamus alternatus* (Coleoptera: Cerambycidae): temperature-dependent development, oviposition and phenology modeling. Ph.D dissertation, Jeju National University, Jeju, Korea.

Lin, S., Hudson, A.C., Richards, A.G., 1954. An analysis of threshold temperatures for the development of *Oncopeltus* and *Tribolium* eggs. Phys. Zool. 27, 287-310.

Rassati, D., Lieutier, F., Faccoli, M., 2016. Alien wood-boring beetles in Mediterranean regions. Paine, T.D., Lieutier, F. (eds.), In: Insects and diseases of Mediterranean forest systems. pp. 293-326. Springer International Publishing, Switzerland.

SAS Institute. 2000. SAS system for window, release 9.4. SAS Institute, Cary, NC.

Togashi, K., 1989a. Development of *Monochamus alternatus* Hope (Coleoptera: Cerambycidae) in relation to oviposition time. Japanese Journal of Applied Entomolgyand Zoology 33, 1-8.

Togashi, K., 1989b. Development of *Monochamus alternatus* Hope (Coleoptera: Cerambycidae) in *Pinus thunbergii* trees weakened at different times. Journal of the Japanese Forestry Society 71, 383-386.

Togashi, K., 1991. Different development of overwintering larvae of *Monochamus alternatus* (Coleoptera: Cerambycidae) under a constant temperature. Jpn. J. Entomol. 59, 149-154.

Supplementary Information III. Field examples of the four instar pathway selected in cool habitat environment

Monochamus alternatus can overwinter as a form of all stages of instars (Togashi, 1989b). The final instar larvae that have finished diapause are directly pupated without resuming food intake (feeding), and emerge as adults in the following spring (Togashi, 1989a, 1989b). Cold temperatures during the winter are the cue to terminate the diapause (Togashi, 1991a; 1991b). Therefore, *M. alternatus* needs additional degree days (DDs) to develop into the fourth or fifth instar, as well as the degree days for larval development as provided in Suppl. I Table S1; namely degree days for the development from post-diapause larvae to adult emergence, pre-oviposition of females, and the hatch of eggs. In regard to this parameter, 1,369 DD and 1,682 DD based on 12.4 °C were suggested for the completion of development to the fourth instar and fifth instar, respectively (JSSGP, 2020).

Suppl. II Fig. S3 presents the study sites of Kojima and Katagiri (1964), Togashi (1989b), Liu et al. (2008), Guo et al. (2015) and the present study. The authors reported the final instar larvae with the fourth or fifth instars according to the study sites (Suppl. II Table S6). The average temperatures for 30 years from 1970 to 2000 were obtained from the data sets of WorldClim Version 2.0 (http://worldclim.org/; Fick and Hijmans, 2017). These climatic data provided annual mean temperature and minimum temperature of coldest month in the study sites. The annual mean temperature and minimum temperature of coldest month were apparently low in the study sites of Togashi (1989b), where the four instar pathway was observed. On the other hand, the weather variables were somewhat higher in the study sites of Kojima and Katagiri (1964), Liu et al. (2008), Guo et al. (2015), and the present study, than those of Togashi (1989b), in which five instar pathway was confirmed. These field observations indicate that the four instar pathway was selected in cool environment.

The weather data for the calculation of degree days in the study years were obtained from the nearest weather station at each study site. The degree days were accumulated from the 1st January based on 12.4 °C. In the study sites of Togashi (1989b), (1,443 to 1,682) DD were accumulated in Hodatsusimizu, and (1,267 to 1,418) DD in Tatsunokuchi, depending on year, Japan (Suppl. II Table S6). Therefore, most larvae would not reach the fifth

instars in Tatsunokuchi and Oshimizu, resulting in the selection of four instar pathway, because the criteria for the completion of development to the fourth instar and fifth instar are 1,369 DD and 1,682 DD, respectively. Namely, only a few larvae that were very early born would be able to develop to the fifth instar.

However, degree days of >2,000 DD were accumulated in the study sites of Kojima and Katagiri (1964) and the present study. In the study sites of Liu et al. (2008) and Guo et al. (2015) in China, degree days would exceed 2,000 DD, since the annual mean temperature was higher than in Jeju. Thus in these regions, a number of larvae would reach to the fifth instar, resulting in the selection of five instar pathway.

Supplementary Table S6. Climatic comparison among the study sites of Kojima and Katagiri (1964), Togashi (1989b), Liu et al. (2008), Guo et al. (2015) and the present study (see Fig. S3 for the locations on map). The accumulated degree days based on 12.4 °C (see Suppl. I Table S1) for full seasons were calculated in the study years.

Country, Researcher	Study sites	Recorded final instar number	Degree days in studied year		Temperature, °C (Avg. 30 yr)	
			Year	Accumulated	Ann. mean	Min. coldest month
Japan, Togashi (1989b)	Oshimizu in Ishikawa (Coastal area)	Four	1979	1,682	13.6	-1.1
			1980	1,478		
			1981	1,443		
	Tatsunokuchi in Ishikawa (Mountainous area, 140 m)	Four	1979	1,418	12.7	-2.9
			1980	1,267		
			1981	1,292		
Japan, Kojima and Katagiri (1964)	Kochi	Five	1963	2,122	16.8	3.2
China, Liu et al. (2008)	Yuyao	Five	Not shown	-	16.8	1.7
China, Guo et al (2015)	Changdong	Four, Five	2014	-[1]	16.0	-0.3
Korea, this study	Jeju	Four, Five	2018	2,204	15.6	2.2

[1]Weather data are not available in the year.

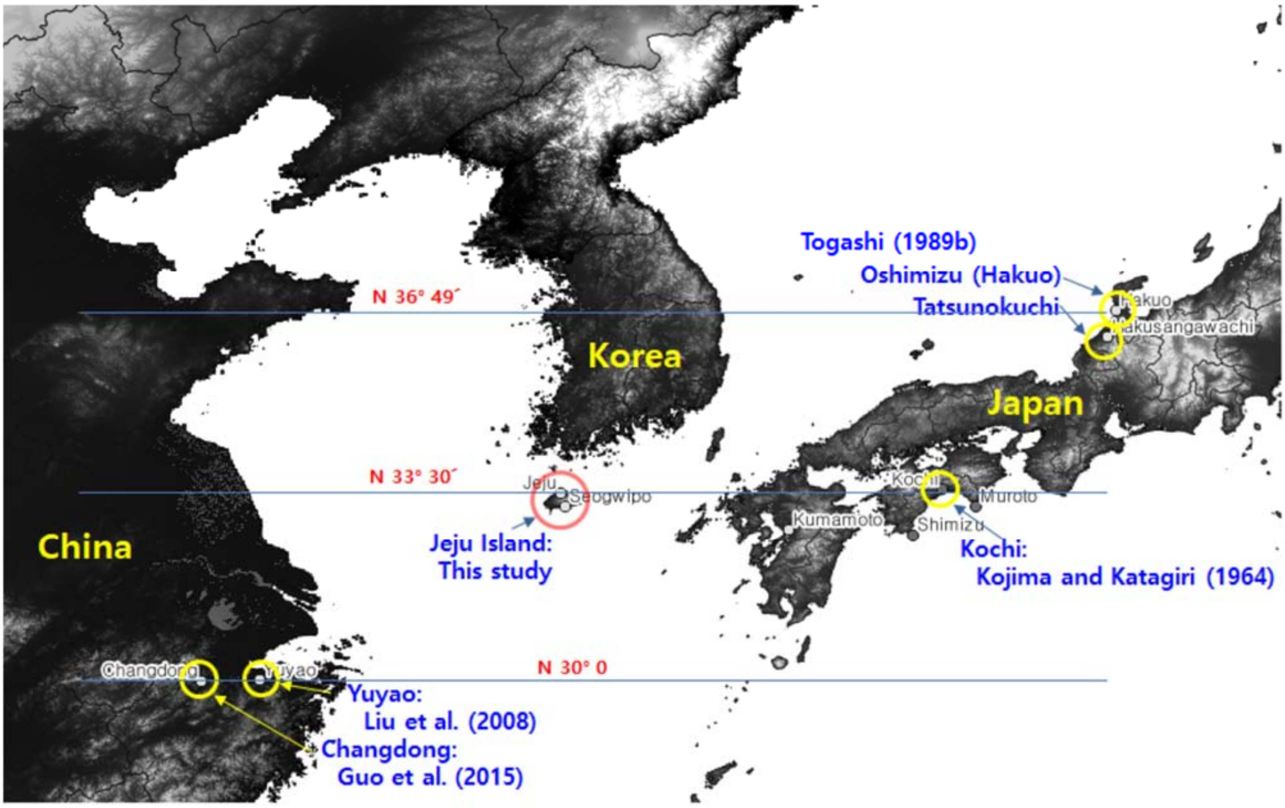

Supplementary Figure S3. Study locations for the instar number of *M. alternatus* in Korea, China, and Japan, showing different latitude. The climatic comparison is seen in Table S6 among the study sites of Kojima and Katagiri (1964), Togashi (1989b), Liu et al. (2008), Guo et al. (2015), and the present study.

References (Suppl. III)

Fick, S.E. and Hijmans, R.J., 2017. WorldClim 2: new 1-km spatial resolution climate surfaces for global land areas. Int. J. Climatol. 37, 4302-4315.

Guo, Y.-Q., Xin, Y.-C., Tao, X., Yu, H., Liu, Z.-D., 2015. Body size difference of male and female adults as well as the relationship between the sizes of pupae and adults and the body weight of overwintering larvae in the Japanese pine sawyer, Monochamus alteratus (Coleoptera: Cerambycidae). Acta Entomologica Sinica 58, 989-996.

JSSGP (Jeju Special Self-Governing Province), 2020. Manual for the control of pine wilt disease in Jeju areas. Publication No. 79-6500000-000461-14. (in Korean)

Kojima, K., Katagiri, K., 1964. On the larval instar and changes of its composition of Hope. J. Jpn. For. Soc. 46, 307-310.

Kojima, K., Katagiri, K., 1964. On the larval instar and changes of its composition of Hope. J. Jpn. For. Soc. 46, 307-310.

Liu, J. D., Li, B. W., Wang, J. Y., Zhao, B. A., Shu, J. P., 2008. Primary study on method to determine instars of *Monochamus alternatus* larvae. J. Zhejiang For. Sci. Technol. 28, 84–86.

Togashi, K., 1989a. Development of *Monochamus alternatus* Hope (Coleoptera: Cerambycidae) in relation to oviposition time. Japanese Journal of Applied Entomolgyand Zoology 33, 1-8.

Togashi, K., 1989b. Development of *Monochamus alternatus* Hope (Coleoptera: Cerambycidae) in *Pinus thunbergii* trees weakened at different times. Journal of the Japanese Forestry Society 71, 383-386.

Togashi, K., 1990. Life table for *Monochamus alternatus* (Coleoptera, Cerambycidae) within dead trees of *Pinus thunbergii*. Jpn. J. Entomol. 58, 217-230.

Togashi, K., 1991a. Different developments of overwintered larvae of *Monochamus alternatus* (Coleoptera, Cerambycidae) under a constant temperature. Jpn. J. Entomol. 59, 149-154.

Togashi, K., 1991b. Larval diapause termination of *Monochamus alternatus* (Coleoptera: Cerambycidae) under natural conditions. Appl. Entomol. Zool. 26, 381-386.
